# On the Solvability of the Discrete *s*(*x*,·)-Laplacian Problems on Simple, Connected, Undirected, Weighted, and Finite Graphs

**DOI:** 10.3390/e24101353

**Published:** 2022-09-24

**Authors:** Filip Pietrusiak, Renata Wieteska, Przemysław Gordinowicz

**Affiliations:** 1 The Institute of Mathematics, Lodz University of Technology, Al. Politechniki 8, 93-590 Lodz, Poland; 2 The Center of Education of Mathematics and Physics, Lodz University of Technology, Al. Politechniki 11, 90-924 Lodz, Poland

**Keywords:** simple graph, connected graph, undirected graph, weighted graph, finite graph, *s*(*x*,·)-Laplacian on a graph, monotonicity methods, positive solutions

## Abstract

We use the finite dimensional monotonicity methods in order to investigate problems connected with the discrete $s\left(x,\cdot \right)$-Laplacian on simple, connected, undirected, weighted, and finite graphs with nonlinearities given in a non-potential form. Positive solutions are also considered.

## 1. Introduction

In this work, we are concerned with the existence and uniqueness of results to discrete, nonlinear equations governed by the variable Laplacian and considered on a finite connected graph. The methods which we apply pertain to the finite dimensional version of the monotonicity theory and therefore rely on coercivity, monotonicity, and continuity of the relevant operators which may contain non-potential terms. Contrary to what is known about problems with variable exponents in the discrete setting (see for example [1,2]), the analysis related to the coercivity of the difference operator must be performed with some care. This is due to the structure of the domain (compare with [3,4] where problems with constant exponent are considered). Another problem appears with the proper definition of the weak solution, which must take into account both the graph structure of the domain and also the fact that the exponent is now not constant. Problems arising here require analysis related to some symmetry. Having overcome the problems mentioned, one may resort to checking the existence of solutions to abstract tools known from the applied functional analysis. In this work, we have decided to employ the monotonicity theory, but one expects that other approaches would also be applicable. In addition, we indicate that a variational approach can be employed in case the given problem is potential. However, the growth assumptions would, in such a case, be similar to those used in the monotonicity approach, and this is why we do not directly formulate such results. When checking the solvability of problems on graphs one is also interested in some well-posedness and stability, structural stability including the following.

Let G=(V,E,ω) be a simple, connected, undirected, weighted, and finite graph. Set *V* is a non-empty set of vertices, and $E\subset {\left[V\right]}^{2}$ is a set consisting of all graph edges, i.e., unordered pairs elements of *V*. Function ω:V×V→[0,+∞) is a weight on a graph *G*, i.e., we assume that

(i) $\forall_{x,y\in V}:\omega \left(x,y\right)=\omega \left(y,x\right)$,
(ii) $\forall_{x,y\in V}:\omega \left(x,y\right)=0\Leftrightarrow \left\{x,y\right\}\notin E$.

Let $f:V\times R\times R^{\left|V\right|}\to R$ be a continuous function, q:V→(0,+∞), p∈[2;+∞),u∈V→R, s:V×V→[2,+∞); moreover, we assume that s(x,y)=s(y,x) for all x,y∈V. Then we consider the following problem:

(1) $$-\Delta_{s(x,\cdot )}u\left(x\right)+q\left(x\right){\left|u(x)\right|}^{p}u\left(x\right)=f\left(x,u\left(x\right),\nabla_{s(x,\cdot )}u\left(x\right)\right),$$

for x∈V. Our goal is to find sufficient conditions for the existence and uniqueness of the solution to (1) in the space

A:={u:V→R}.

Note that *A* is a |V|-dimensional Euclidean space with the norm given by

(2) $$∥u∥:={\left(\sum_{x\in V}{\left|u(x)\right|}^{2}\right)}^{\frac{1}{2}}.$$

Now we define two operators, which are necessary in order to properly understand our problem.

**Definition** **1.** *Let u:V→R and s:V×V→[2,+∞).*

(i) *The s(x,·)-gradient of the function u in point x∈V, $\nabla_{s(x,\cdot )}:A\to R^{\left|V\right|}$, is defined by*
(3) $$\nabla_{s(x,\cdot )}u\left(x\right):={\left(D_{s(x,y)}u\left(x\right)\right)}_{y\in V}\;\;,$$
*where*
(4) $$D_{s(x,y)}u\left(x\right):={\left|u(y)-u(x)\right|}^{s(x,y)-2}\left(u\left(y\right)-u\left(x\right)\right)\sqrt{\omega (x,y)},$$
*for all x∈V, is called the s(·,·)-directional derivative of the function u in the direction y∈V. In the case where s(·,·)≡2, we write ∇.*
(ii) *The discrete s(x,·)-Laplacian of the function u at point x∈V, $\Delta_{s(x,\cdot )}:A\to R$, is defined by*
(5) $$\begin{array}{cc} \Delta_{s(x,\cdot )}u\left(x\right):= & \sum_{y\in V}{\left|u(y)-u(x)\right|}^{s(x,y)-2}\left(u\left(y\right)-u\left(x\right)\right)\omega \left(x,y\right)\\ = & \sum_{y\in V}D_{s(x,y)}u\left(x\right)\sqrt{\omega (x,y)}. \end{array}$$
  *If s(·,·)≡2 we write* Δ.

On the right hand side of Formula (3) there appears a |V|-dimensional vector that is indexed by y∈V. We would like to underline that to the best of our knowledge, the discrete s(x,·)-Laplacian on finite graphs have not been considered yet, which means that several auxiliary tools that are known on a graph setting must be worked out in detail. This means that we had to investigate the problem in a detailed manner which involves derivation of some auxiliary tools. The results in the literature cover only the case of the *p*-Laplacian and $p\left(x\right)$-Laplacian on graphs, where, however, other methods are applied and monotonicity approaches are not used. Thus, our results are also new in the context of constant p. In case s(·,·) is a constant function, we may recover the well-known *p*-Laplacian on a graph setting for which there are some existence and multiplicity results (see [5]).

The graph s(x,·)-Laplacian correspond to anisotropic boundary value problems which on the other hand serve as mathematical models used in elastic mechanics [6] or image restoration [7]. Variational continuous anisotropic problems have been started by Fan and Zhang in [8]. A very extensive study contained in [9] provides many tools and methods used in the area of anisotropic problems. The general reference for discrete problems is [10], whereas for the background in graph theory we refer to [11]. The existence of positive solutions follows the pattern employed for problems with constant *p*.

The paper is organized as follows. We start with a summation-by-parts formula and some inequalities that are used further on. The summation-by-parts formula is used in derivation of a notion of a weak solution, whereas relevant inequalities are used in order to apply monotonicity techniques. However, these may useful also when applying other nonlinear analysis tools. Next, existence and uniqueness is concerned. Finally the question of the existence of positive solutions is undertaken.

## 2. Preliminary and Auxiliary Results

For the weak formulation of problem (1), we need the following lemma, which is in fact a graph variant of summation by parts

**Lemma** **1.** *For any pair of functions u,v:V→R, if $s\left(x,y\right)=s\left(y,x\right)$ for all x,y∈V, we have*

$$-2\sum_{x\in V}(\Delta_{s(x,\cdot )}u\left(x\right))v\left(x\right)=\sum_{x\in V}\langle \nabla_{s(x,\cdot )}u\left(x\right),\nabla v\left(x\right)\rangle ,$$

*where 〈·,·〉 is a Euclidean scalar product in $R^{\left|V\right|}$.*

**Proof.**  Let u,v:V→R. By Formulas (3)–(5) we have

$$\begin{array}{cc} \sum_{x\in V}\langle \nabla_{s(x,\cdot )}u\left(x\right),\nabla v\left(x\right)\rangle = & \sum_{x,y\in V}{\left|u(y)-u(x)\right|}^{s(x,y)-2}\left(u\left(y\right)-u\left(x\right)\right)\omega \left(x,y\right)\left(v\left(y\right)-v\left(x\right)\right)\\ = & \sum_{x,y\in V}{\left|u(y)-u(x)\right|}^{s(x,y)-2}\left(u\left(y\right)-u\left(x\right)\right)\omega \left(x,y\right)v\left(y\right)\\ & -\sum_{x,y\in V}{\left|u(y)-u(x)\right|}^{s(x,y)-2}\left(u\left(y\right)-u\left(x\right)\right)\omega \left(x,y\right)v\left(x\right)\\ = & \sum_{x,y\in V}{\left|u(x)-u(y)\right|}^{s(y,x)-2}\left(u\left(x\right)-u\left(y\right)\right)\omega \left(y,x\right)v\left(x\right)\\ & -\sum_{x\in V}(\Delta_{s(x,\cdot )}u\left(x\right))v\left(x\right)\\ = & -\sum_{x,y\in V}{\left|u(y)-u(x)\right|}^{s(x,y)-2}\left(u\left(y\right)-u\left(x\right)\right)\omega \left(x,y\right)v\left(x\right)\\ & -\sum_{x\in V}(\Delta_{s(x,\cdot )}u\left(x\right))v\left(x\right)\\ = & -2\sum_{x\in V}(\Delta_{s(x,\cdot )}u\left(x\right))v\left(x\right). \end{array}$$

We want to emphasize that the symmetry of function *s* is obligatory here, because we need equality ${\left|u(x)-u(y)\right|}^{s(y,x)-2}={\left|u(y)-u(x)\right|}^{s(x,y)-2}$. □

Next, we will derive a weak formulation of problem (1). Multiplying Equation (1) by some v∈A, and then by taking summation on both sides and using Lemma 1, we get

(6) $$\begin{array}{cc} \; & \frac{1}{2}\sum_{x\in V}\langle \nabla_{s(x,\cdot )}u\left(x\right),\nabla v\left(x\right)\rangle +\sum_{x\in V}q\left(x\right){\left|u(x)\right|}^{p}u\left(x\right)v\left(x\right)\\ \; & =\sum_{x\in V}v\left(x\right)f(x,u\left(x\right),\nabla_{s(x,\cdot )}u\left(x\right)). \end{array}$$

If the above equation is fulfilled for all v∈A, then it is equivalent to having Equation (1) for all x∈V. To prove that, for each $x_{0}\in V$, we take $v=\delta_{x_{0}}$, where

$$\delta_{x_{0}}\left(x\right)=\left\{\begin{array}{cc} 1, & \mathrm{when}\;x=x_{0},\\ 0, & \mathrm{when}\;x\neq x_{0}. \end{array}\right.$$

This implies (1) for all x∈V.

Let us also introduce a notation used throughout the paper, namely

$$\begin{array}{cc} s^{-}:=\min_{x,y\in V}s(x,y), & s^{+}:=\max_{x,y\in V}s(x,y),\\ q^{+}:=\max_{x\in V}q\left(x\right), & \omega^{+}:=\max_{x,y\in V}\omega (x,y). \end{array}$$

For the case of our problem, we need several inequalities known from [12]. We provide the suitable proofs due to many subtle differences. These differences arise from the fact of how we treat the boundary of the graph. This is in contrast to [12] where the boundary plays its role, and therefore there appear several restrictions with which we do not need to cope.

**Lemma** **2.** *On the space A the following inequalities hold.*

*(i)*  *For every u∈A and for every m≥0 we have*
$$\sum_{x\in V}{\left|u\left(x\right)\right|}^{m}\leq \left|V\right|{∥u∥}^{m}.$$
*(ii)*  *For every u∈A and for every m≥2 we have*
$$\sum_{x,y\in V}{\left|u(y)-u(x)\right|}^{m}\leq 2^{m}{\left|V\right|}^{2}{∥u∥}^{m}.$$
*(iii)*  *Let $s^{+}\geq 2$ for every u∈A, and we have*
$$\sum_{x,y\in V}{\left|u(y)-u(x)\right|}^{s(x,y)}\omega \left(x,y\right)\leq \omega^{+}2^{s^{+}}{\left|V\right|}^{2}{∥u∥}^{s^{+}}+\omega^{+}{\left|V\right|}^{2}.$$
*(iv)*  *For every u∈A and for every m≥2 we have*
$$\sum_{x\in V}{\left|u(x)\right|}^{m}\geq {\left|V\right|}^{\frac{2-m}{2}}{∥u∥}^{m}.$$

**Proof.**  To see (i), note that for any fixed x∈V we have

$${\left|u\left(x\right)\right|}^{2}\leq \sum_{y\in V}{\left|u\left(y\right)\right|}^{2},$$

so

$$\left|u\left(x\right)\right|\leq ∥u∥.$$

Therefore for every m≥0 we obtain

$$\sum_{x\in V}{\left|u\left(x\right)\right|}^{m}\leq \left|V\right|{∥u∥}^{m}.$$

Relation (ii) is obtain from the triangle inequality and from inequality (i), namely

$$\begin{array}{cc} \sum_{x,y\in V}{\left|u(y)-u(x)\right|}^{m} & \leq \sum_{x,y\in V}{\left(\left|u(y)\right|+\left|u(x)\right|\right)}^{m}\\ \; & \leq \sum_{x,y\in V}{\left(2∥u∥\right)}^{m}=2^{m}{\left|V\right|}^{2}{∥u∥}^{m}. \end{array}$$

At first with inequality (iii), the most important thing is to divide sum with respect to the 1, to effectively use values of $s^{-}$ and $s^{+}$

$$\begin{array}{cc} \sum_{x,y\in V}{\left|u(y)-u(x)\right|}^{s(x,y)}\omega \left(x,y\right)\leq & \omega^{+}\sum_{x,y\in V}{\left|u(y)-u(x)\right|}^{s(x,y)}\\ = & \omega^{+}\sum_{\left\{x,y\in V:\left|u\left(y\right)-u\left(x\right)\right|\leq 1\right\}}{\left|u(y)-u(x)\right|}^{s(x,y)}\\ \; & +\omega^{+}\sum_{\left\{x,y\in V::\left|u\left(y\right)-u\left(x\right)>1\right|\right\}}{\left|u(y)-u(x)\right|}^{s(x,y)}\\ \leq & \omega^{+}\sum_{\left\{x,y\in V::\left|u\left(y\right)-u\left(x\right)\right|\leq 1\right\}}{\left|u(y)-u(x)\right|}^{s^{-}}\\ \; & +\omega^{+}\sum_{\left\{x,y\in V::\left|u\left(y\right)-u\left(x\right)>1\right|\right\}}{\left|u(y)-u(x)\right|}^{s^{+}}\\ = & \omega^{+}\sum_{\left\{x,y\in V::\left|u\left(y\right)-u\left(x\right)\right|\leq 1\right\}}\left({\left|u(y)-u(x)\right|}^{s^{-}}-{\left|u(y)-u(x)\right|}^{s^{+}}\right)\\ \; & +\omega^{+}\sum_{x,y\in V}{\left|u(y)-u(x)\right|}^{s^{+}}. \end{array}$$

Now we can use inequality (ii) to get the following:

$$\sum_{x,y\in V}{\left|u(y)-u(x)\right|}^{s^{+}}\leq 2^{s^{+}}{\left|V\right|}^{2}{∥u∥}^{s^{+}}.$$

Moreover,

$$\sum_{\left\{x,y\in V::\left|u\left(y\right)-u\left(x\right)\right|\leq 1\right\}}\left({\left|u(y)-u(x)\right|}^{s^{-}}-{\left|u(y)-u(x)\right|}^{s^{+}}\right)\leq \sum_{\left\{x,y\in V::\left|u\left(y\right)-u\left(x\right)\right|\leq 1\right\}}1\leq {\left|V\right|}^{2}.$$

Consequently, we finally have

$$\sum_{x,y\in V}{\left|u(y)-u(x)\right|}^{s(x,y)}\omega \left(x,y\right)\leq \omega^{+}2^{s^{+}}{\left|V\right|}^{2}{∥u∥}^{s^{+}}+\omega^{+}{\left|V\right|}^{2}.$$

We will show that (iv) holds. First, we will use discrete Hölder’s inequality with $p=\frac{m}{2}$ and $q=\frac{m}{m-2}$ for m>2

$$\sum_{x\in V}1\cdot {\left|u(x)\right|}^{2}\leq {\left(\sum_{x\in V}1^{\frac{m}{m-2}}\right)}^{\frac{m-2}{m}}{\left(\sum_{x\in V}{\left|u\left(x\right)\right|}^{m}\right)}^{\frac{2}{m}}.$$

Then we use simple transformations to get our inequality

$$\begin{array}{cc} {∥u∥}^{2} & \leq {\left|V\right|}^{\frac{m-2}{m}}{\left(\sum_{x\in V}{\left|u\left(x\right)\right|}^{m}\right)}^{\frac{2}{m}},\\ ∥u∥ & \leq {\left|V\right|}^{\frac{m-2}{2m}}{\left(\sum_{x\in V}{\left|u\left(x\right)\right|}^{m}\right)}^{\frac{1}{m}} \end{array}$$

and finally

$$\sum_{x\in V}{\left|u(x)\right|}^{m}\geq {\left|V\right|}^{\frac{2-m}{2}}{∥u∥}^{m}.$$

In the end, we notice that for m=2 we get

$$\begin{array}{cc} \sum_{x\in V}{\left|u(x)\right|}^{2} & \geq {\left|V\right|}^{0}{∥u∥}^{2}\\ {∥u∥}^{2} & \geq 1\cdot {∥u∥}^{2}, \end{array}$$

so it works also for m=2. □

**Lemma** **3** (see [13] p. 3). *Let n∈N, $a,b\in R^{n}$ and p≥2. Then we have the following inequality*

$$\langle {∥a∥}^{p-2}a-{∥b∥}^{p-2}b,:a-b\rangle \geq c_{p}∥a-b∥,$$

*where $c_{p}=\frac{2}{p\left(2^{p-1}-1\right)}$; moreover $\langle \cdot ,\cdot \rangle$ denotes an inner scalar product in $R^{n}$ and $∥\cdot ∥$ denotes a Euclidean norm in $R^{n}$.*

The methods which we employ rely on monotonicity notions (see [14]). By *A* we denote the finite dimensional real Banach space, by $A^{*}$ its dual, $\langle \cdot ,\cdot \rangle$ denotes the duality pairing between $A^{*}$ and *A*, i.e., the scalar product.

**Definition** **2.** Operator $T:A\to A^{*}$ is called

*(i)*  *bounded if it maps bounded sets into bounded sets,*
*(ii)*  *coercive if*
$$\lim_{{∥u∥}_{A}\to +\infty }\frac{\langle T(u),u\rangle }{{∥u∥}_{A}}\to +\infty ,$$
*(iii)*  *monotone if*
$$\langle T\left(u\right)-T\left(v\right),u-v\rangle \geq 0,$$
*for all u,v∈A, and (iv) strictly monotone if*
$$\langle T\left(u\right)-T\left(v\right),u-v\rangle >0,$$
*for any u≠v.*

**Theorem** **1.** Let $T:A\to A^{*}$ be continuous and coercive. Then T is surjective, i.e., for any $f\in A^{*}$, there is at least one solution to the equation Tu=f. If A is additionally strictly monotone, then A is a homeomorphism.

## 3. Existence and Uniqueness of Solutions

In order to use the monotonicity tools, we introduce the following operator $T:A\to A^{*}$

$$\langle T(u),v\rangle :=\langle T_{1}\left(u\right),v\rangle +\langle T_{2}\left(u\right),v\rangle -\langle T_{3}\left(u\right),v\rangle ,$$

where

$$\begin{array}{cc} \langle T_{1}\left(u\right),v\rangle & :=:\frac{1}{2}\sum_{x\in V}\langle \nabla_{s(x,\cdot )}u\left(x\right),\nabla v\left(x\right)\rangle ,\\ \langle T_{2}\left(u\right),v\rangle & :=\sum_{x\in V}q\left(x\right){\left|u(x)\right|}^{p}u\left(x\right)v\left(x\right),\\ \langle T_{3}\left(u\right),v\rangle & :=\sum_{x\in V}v\left(x\right)f(x,u\left(x\right),\nabla_{s(x,\cdot )}u\left(x\right)). \end{array}$$

We observe that when if for u∈A it follows that

$$\langle T(u),v\rangle =0\;\mathrm{for}\;\mathrm{all}\;v\in A$$

then *u* is a solution to (1).

**Lemma** **4.** *(i) If for all u∈R and for all $v\in R^{\left|V\right|}$ we have*

(7) $$uf\left(x,u,v\right)\leq {\left|u\right|}^{\alpha_{1}}+\beta_{1},$$

*where $\alpha_{1}<p+2$, $\beta_{1}\in R$, then operator T is coercive, and (ii) if for all u∈R and for all $v\in R^{\left|V\right|}$, we have*

(8) $$uf\left(x,u,v\right)\geq {\left|u\right|}^{\alpha_{2}}+\beta_{2},$$

*where $\alpha_{2}>\max\left(s^{+},p+2\right)$, $\beta_{2}\in R$, and then operator −T is coercive.*

**Proof.**  In the first case, we want to find estimate for the operators $T_{1}$, $T_{2}$ from the bottom. From the definition of $T_{1}$ we have

$$\langle T_{1}\left(u\right),u\rangle =\frac{1}{2}\sum_{x,y\in V}{\left|u(y)-u(x)\right|}^{s(x,y)}\omega \left(x,y\right)\geq 0.$$

The second estimate by (iv) from Lemma 2

$$\begin{array}{cc} \langle T_{2}\left(u\right),u\rangle = & \sum_{x\in V}q\left(x\right){\left|u(x)\right|}^{p+2}\geq q^{-}\sum_{x\in V}{\left|u(x)\right|}^{p+2}\\ \geq & q^{-}{\left|V\right|}^{\frac{-p}{2}}{∥u∥}^{p+2}. \end{array}$$

Hence we have

$$\langle T_{1}\left(u\right),u\rangle +\langle T_{2}\left(u\right),u\rangle \geq q^{-}{\left|V\right|}^{\frac{-p}{2}}{∥u∥}^{p+2}.$$

Moreover, from (7) we obtain

$$\langle T_{3}\left(u\right),u\rangle =\sum_{x\in V}u\left(x\right)f(x,u\left(x\right),\nabla_{s(x,\cdot )}u\left(x\right))\leq \left|V\right|{∥u∥}^{\alpha_{1}}+\left|V\right|\beta_{1}.$$

Finally, we see that

$$\langle T(u),u\rangle \geq q^{-}{\left|V\right|}^{\frac{-p}{2}}{∥u∥}^{p+2}-\left|V\right|{∥u∥}^{\alpha_{1}}-\left|V\right|\beta_{1}$$

because $\alpha_{1}<p+2$ operators $T_{1}$ and $T_{2}$ dominate, and it is easy to see that operator *T* is coercive.

In the second case, we want to find an estimate for the operators $T_{1}$, $T_{2}$ from the above. First, estimate by (iii) from Lemma 2

$$\begin{array}{cc} \langle T_{1}\left(u\right),u\rangle & =\frac{1}{2}\sum_{x,y\in V}{\left|u(y)-u(x)\right|}^{s(x,y)}\omega \left(x,y\right)\leq \\ \; & \leq \omega^{+}2^{s^{+}-1}{\left|V\right|}^{2}{∥u∥}^{s^{+}}+\frac{1}{2}\omega^{+}{\left|V\right|}^{2}. \end{array}$$

Secondly, estimate by (i) from Lemma 2

$$\langle T_{2}\left(u\right),u\rangle =\sum_{x\in V}q\left(x\right){\left|u(x)\right|}^{p+2}\leq q^{+}\left|V\right|{∥u∥}^{p+2}.$$

Now we see that

$$\langle T_{1}\left(u\right),u\rangle +\langle T_{2}\left(u\right),u\rangle \leq a{∥u∥}^{\max\left(s^{+},p+2\right)}+b,$$

where

$$a=\omega^{+}2^{s^{+}-1}{\left|V\right|}^{2}+q^{+}\left|V\right|$$

and

$$b=\frac{1}{2}\omega^{+}{\left|V\right|}^{2}.$$

Moreover, from (8) we have

$$\begin{array}{cc} \langle T_{3}\left(u\right),u\rangle = & \sum_{x\in V}u\left(x\right)f(x,u\left(x\right),\nabla_{s(x,\cdot )}u\left(x\right))\geq \sum_{x\in V}\left({\left|u\left(x\right)\right|}^{\alpha_{2}}+\beta_{2}\right)\\ \; & =\sum_{x\in V}{\left|u\left(x\right)\right|}^{\alpha_{2}}+\left|V\right|\beta_{2}\geq {\left|V\right|}^{\frac{2-\alpha_{2}}{2}}{∥u∥}^{\alpha_{2}}+\left|V\right|\beta_{2}. \end{array}$$

Finally, we see that

$$\langle -T(u),u\rangle \geq {\left|V\right|}^{\frac{2-\alpha_{2}}{2}}{∥u∥}^{\alpha_{2}}+\left|V\right|\beta_{2}-a{∥u∥}^{\max\left(s^{+},p+2\right)}-b.$$

Because $\alpha_{2}>\max\left(s^{+},p+2\right)$ operator $T_{3}$ dominates, and it is easy to see that oparator −T is coercive. □

Now we give an example to illustrate condition (7).

**Example** **1.** *Let $f:V\times R\times R^{\left|V\right|}\to R$ and p≥2. Put*

$$f\left(x,t_{1},t_{2}\right)=t_{1}\sin t_{1}-t_{1}t_{2}^{2}e^{-x}+\frac{t_{1}}{1+t_{1}^{2}}.$$

*Observe that*

$$t_{1}f\left(x,t_{1},t_{2}\right)=t_{1}^{2}\sin t_{1}-t_{1}^{2}t_{2}^{2}e^{-x}+\frac{t_{1}^{2}}{1+t_{1}^{2}}$$

*and*

$$t_{1}f\left(x,t_{1},t_{2}\right)\leq {\left|t_{1}\right|}^{2}+1,$$

*so condition (7) is satisfied with $\alpha_{1}=2$$\left(\alpha_{1}<p+2\right)$ and $\beta_{1}=1.$*

To illustrate condition (8) let us take the following example.

**Example** **2.** *Let $f:V\times R\times R^{\left|V\right|}\to R$, s:V×V→[2,10) and p∈[2;8). Put*

$$f\left(x,t_{1},t_{2}\right)=t_{1}^{9}\left(\cos t_{1}+2\right)+t_{1}t_{2}^{2}\mathrm{arccot}x-\frac{3t_{1}}{1+t_{1}^{2}}.$$

*Observe that*

$$t_{1}f\left(x,t_{1},t_{2}\right)=t_{1}^{10}\left(\cos t_{1}+2\right)+t_{1}^{2}t_{2}^{2}\mathrm{arccot}x-\frac{3t_{1}^{2}}{1+t_{1}^{2}}$$

*and*

$$t_{1}f\left(x,t_{1},t_{2}\right)\geq {\left|t_{1}\right|}^{10}-3,$$

*so condition (7) is satisfied with $\alpha_{2}=10\;(\alpha_{2}>\max\left(s^{+},p+2\right)$ and $\beta_{1}=-3.$*

**Theorem** **2.** *If assumption* (7) *or* (8) *holds, then problem* (1) *has at least one solution.*

**Proof.**  Lemma 3 implies that *T* or −T is coercive. Moreover *T* and −T are continuous, so the assumptions of Theorem 1 are fulfilled. This means that there exists a solution to problem (6) that is equivalent to the existence of the solution to problem (1). □

We can observe that the operators $T_{1},T_{2},T_{3}$ have the following properties.

**Lemma** **5.** *(i) Operator $T_{1}$ is monotone, (ii) Operator $T_{2}$ is strictly monotone, (iii) if we assume that the continuous function f does not depend on $\nabla_{s\left(x,\cdot \right)}$, so f:V×R→R and satisfies*

(9) $$\left(u-v\right)\left(f\left(x,u\right)-f\left(x,v\right)\right)\leq 0,$$

*for all x∈V and all u, v∈R, then operator $-T_{3}$ is monotone.*

**Proof.** 

1. By direct calculations and by Lemma 3, we get
  $$\begin{array}{cc} & \langle T_{1}\left(u\right)-T_{1}\left(v\right),u-v\rangle \\ & =\sum_{x,y\in V}\left({\left|u(y)-u(x)\right|}^{s(x,y)-2}\left(u(y)-u(x)\right)-{\left|v(y)-v(x)\right|}^{s(x,y)-2}\left(v(y)-v(x)\right)\right)\left(\left(u(y)-u(x)\right)-\left(v(y)-v(x)\right)\right)\\ & \geq \sum_{x,y\in V}\frac{2}{s\left(x,y\right)\left(2^{s(x,y)-1}-1\right)}\cdot {\left|\left(u(y)-u(x)\right)-\left(v(y)-v(x)\right)\right|}^{s\left(x,y\right)}\geq 0. \end{array}$$
2. By direct calculations and by Lemma 3, we also have
  $$\begin{array}{cc} & \langle T_{2}\left(u\right)-T_{2}\left(v\right),u-v\rangle \\ & =\sum_{x\in V}q\left(x\right)\left({\left|u(x)\right|}^{p}u\left(x\right)-{\left|v(x)\right|}^{p}v\left(x\right)\right)\left(u\left(x\right)-v\left(x\right)\right)\\ & \geq \sum_{x\in V}\frac{2q\left(x\right)}{p\left(2^{p-1}-1\right)}\cdot {\left|u(x)-v(x)\right|}^{p}>0,\;\mathrm{for}\;\mathrm{all}\;u\neq v. \end{array}$$
3. By assumption (9)
  $$\begin{array}{cc} & \langle T_{3}\left(u\right)-T_{3}\left(v\right),u-v\rangle \\ & =\sum_{x\in V}\left(u\left(x\right)-v\left(x\right)\right)\left(f\left(x,u\left(x\right)\right)-f\left(x,v\left(x\right)\right)\right)\leq 0, \end{array}$$
  so $-T_{3}$ is monotone.

□

**Theorem** **3.** *If assumption* (7) *or* (8) *holds, and moreover assumption* (9) *is fulfilled, then problem* (1) *has a unique solution.*

**Proof.**  By direct calculations and by Lemma 3,

$$\begin{array}{cc} \; & \langle T_{1}\left(u\right)-T_{1}\left(v\right),u-v\rangle \\ \; & =\sum_{x,y\in V}\left({\left|u(y)-u(x)\right|}^{s(x,y)-2}\left(u(y)-u(x)\right)-{\left|v(y)-v(x)\right|}^{s(x,y)-2}\left(v(y)-v(x)\right)\right)\left(\left(u(y)-u(x)\right)-\left(v(y)-v(x)\right)\right)\\ \; & \geq \sum_{x,y\in V}\frac{2}{s\left(x,y\right)\left(2^{s(x,y)-1}-1\right)}\cdot {\left|\left(u(y)-u(x)\right)-\left(v(y)-v(x)\right)\right|}^{s\left(x,y\right)}\geq 0. \end{array}$$

By direct calculations and by Lemma 3,

$$\begin{array}{cc} \; & \langle T_{2}\left(u\right)-T_{2}\left(v\right),u-v\rangle \\ \; & =\sum_{x\in V}q\left(x\right)\left({\left|u(x)\right|}^{p}u\left(x\right)-{\left|v(x)\right|}^{p}v\left(x\right)\right)\left(u\left(x\right)-v\left(x\right)\right)\\ \; & \geq \sum_{x\in V}\frac{2q\left(x\right)}{p\left(2^{p-1}-1\right)}\cdot {\left|u(x)-v(x)\right|}^{p}>0,\;\mathrm{for}\;\mathrm{all}\;u\neq v. \end{array}$$

By assumption (9),

$$\begin{array}{cc} \; & \langle T_{3}\left(u\right)-T_{3}\left(v\right),u-v\rangle \\ \; & =\sum_{x\in V}\left(u\left(x\right)-v\left(x\right)\right)\left(f\left(x,u\left(x\right)\right)-f\left(x,v\left(x\right)\right)\right)\leq 0, \end{array}$$

operator $-T_{3}$ is monotone. □

Now we give an example to illustrate the above theorem.

**Example** **3.** *Let f:V×R→R and p>2. Put*

$$f\left(x,t\right)=-t^{3}e^{-2x}.$$

*Observe that*

$$tf\left(x,t\right)=-t^{4}e^{-2x},$$

*and*

$$tf\left(x,t\right)\leq {\left|t\right|}^{4},$$

*so condition* (7) *is satisfied for all x∈V, and all t∈R with $\alpha_{1}=4$$\left(\alpha_{1}<p+2\right)$ and $\beta_{1}=0.$ Note also that*

$$\left(t_{1}-t_{2}\right)\left(f\left(x,t_{1}\right)-f\left(x,t_{2}\right)\right)=\left(t_{1}-t_{2}\right)\left(-t_{1}^{3}e^{-2x}+t_{2}^{3}e^{-2x}\right)=-e^{-2x}{\left(t_{1}-t_{2}\right)}^{4}\leq 0$$

*for all x∈V and all $t_{1}$, $t_{2}\in R$, so condition* (9) *is satisfied. The assumptions of Theorem 3 are fulfilled, so problem* (1) *has a unique solution with any function q:V→(0,+∞).*

As far as we know, Theorem 2 cannot be obtained by a variational method because the function *f* depends on $\nabla_{s\left(x,\cdot \right)}u\left(x\right)$.

On the other hand, the assertion from Theorem 3 can be obtained by variational methods, with small changes in assumptions.

## 4. Positive Solutions

In this section, we will look for positive solutions to problem (1). By a positive solution to problem (1), we mean the solution, which has only positive values on *V*. Positive solutions to (1) are again investigated in the space *A*, considered with the norm (2).

We introduce the following notion:

$$u_{+}\left(x\right)=\max\left\{u\left(x\right),0\right\},\;u_{-}\left(x\right)=\max\left\{-u\left(x\right),0\right\},\;\mathrm{for}\;\mathrm{all}\;x\in V.$$

It is easy to see that for all x∈V we have

$$\begin{array}{cc} u_{+}\left(x\right), & u_{-}\left(x\right)\geq 0,\\ u_{+}\left(x\right)\cdot & u_{-}\left(x\right)=0,\\ u(x) & =u_{+}\left(x\right)-u_{-}\left(x\right),\\ \left|u(x)\right| & =u_{+}\left(x\right)+u_{-}\left(x\right),\\ \left|u_{+}\left(x\right)\right| & \leq \left|u(x)\right|. \end{array}$$

If we assume that

(10) $$f\left(x,u,v\right)>0,\;\mathrm{for}\;\mathrm{all}\;x\in V,\;u\geq 0\;\mathrm{and}\;\mathrm{all}\;v\in R^{\left|V\right|},$$

then we can define a new problem,

(11) $$\begin{array}{cc} \; & \frac{1}{2}\sum_{x\in V}\langle \nabla_{s(x,\cdot )}u\left(x\right),\nabla v\left(x\right)\rangle +\sum_{x\in V}q\left(x\right){\left|u(x)\right|}^{p}u\left(x\right)v\left(x\right)\\ \; & =\sum_{x\in V}v\left(x\right)f(x,u_{+}\left(x\right),\nabla_{s(x,\cdot )}u\left(x\right)). \end{array}$$

The solution to this problem fulfilled (11) for all v∈A. The only difference between (11) and (6) is the right hand side, i.e., the second argument of *f* in (11) is $u_{+}\left(x\right)$ not u(x).

Let us formulate an auxiliary result which plays an important role in proving all the existence results in this section. This result shows that any solution to (6) is in fact a positive solution and simultaneously it is the positive solution to (1). It may be viewed as a kind of a discrete maximum principle.

**Lemma** **6.** *Assume that* (10) holds, and assume that $u^{*}\in A$ is a solution to problem (11). Then $u^{*}$ is a positive solution to (1)*.*

**Proof.**  A straightforward computation shows that for every x,y∈V and u∈A, we have the following inequality:

(12) $$\begin{array}{cc} u\left(x\right)u_{-}\left(x\right) & =\left(u_{+}\left(x\right)-u_{-}\left(x\right)\right)u_{-}\left(x\right)\\ \; & =u_{+}\left(x\right)u_{-}\left(x\right)-{\left(u_{-}\left(x\right)\right)}^{2}=-{\left(u_{-}\left(x\right)\right)}^{2}\leq 0. \end{array}$$

Moreover,

(13) $$\left(u(y)-u(x)\right)\left(u_{-}\left(y\right)-u_{-}\left(x\right)\right)\leq 0$$

holds, because

$$\begin{array}{cc} \left(u(y)-u(x)\right) & \left(u_{-}\left(y\right)-u_{-}\left(x\right)\right)\\ & =\left(\left(u_{+}\left(y\right)-u_{+}\left(x\right)\right)-\left(u_{-}\left(y\right)-u_{-}\left(x\right)\right)\right)\left(u_{-}\left(y\right)-u_{-}\left(x\right)\right)\\ & =-u_{+}\left(y\right)u_{-}\left(x\right)-u_{+}\left(x\right)u_{-}\left(y\right)-{\left(u_{-}\left(y\right)-u_{-}\left(x\right)\right)}^{2}\leq 0. \end{array}$$

Assume that $u^{*}\in A$ is a solution to (11). We set $v=u_{-}^{*}$, and we get

(14) $$\begin{array}{cc} \; & \frac{1}{2}\sum_{x,y\in V}{\left|u^{*}\left(y\right)-u^{*}\left(x\right)\right|}^{s(x,y)-2}\left(u^{*}\left(y\right)-u^{*}\left(x\right)\right)\left(u_{-}^{*}\left(y\right)-u_{-}^{*}\left(x\right)\right)\omega \left(x,y\right)\\ \; & =\sum_{x\in V}f\left(x,u_{+}^{*}\left(x\right),\nabla_{p(x)}u^{*}\left(x\right)\right)u_{-}^{*}\left(x\right)-\sum_{x\in V}q\left(x\right){\left|u^{*}\left(x\right)\right|}^{p}u^{*}\left(x\right)u_{-}^{*}\left(x\right). \end{array}$$

Because *f* and *q* are functions with positive values only and because (12) holds, the terms on the right hand side of (14) are together non-negative.

Due to (13), the term on the left hand side of (14) is non-positive.

Therefore, Equation (14) holds only if its both sides are equal to zero, which leads to $u_{-}^{*}\left(x\right)=0$ for all x∈V. Thus, $u^{*}\left(x\right)=u_{+}^{*}\left(x\right)$ for all x∈V, which means that for $u^{*}$ problems (6) and (11) are equivalent, so in fact $u^{*}$ is solution of (1).

Moreover, $u^{*}\left(x\right)\neq 0$ for all x∈V. Indeed, assume that there exists $x_{0}\in V$ such that $u^{*}\left(x_{0}\right)=0$. Then, by (1) we have

$$-\sum_{y\in V\setminus \{x_{0}\}}{\left|u^{*}\left(y\right)\right|}^{s(x_{0},y)-1}\omega \left(x_{0},y\right)=f\left(x_{0},0,\nabla_{s(x_{0},.)}u^{*}\left(x_{0}\right)\right).$$

Because the term on the left is non-positive and the term on the right is positive, we have a contradiction. Thus $u^{*}\left(x\right)\neq 0$ for all x∈V, it follows that $u^{*}$ is a positive solution to (1). □

To illustrate assumption (10), take the following example.

**Example** **4.** *Let $f:V\times R\times R^{\left|V\right|}\to R$ and put*

$$f\left(x,t_{1},t_{2}\right)=5t_{1}+e^{t_{2}}+\arccos x.$$

*It is easy to see that*

$$f(x,t_{1},t_{2})>0$$

*for all x∈V, for all $t_{1}\geq 0$ and for all $t_{2}\in R^{\left|V\right|}.$*

**Remark** **1.** *We can easily get analogical results for negativity of solutions, by a change of signs in assumption* (10), *in exactly the same way.*

**Remark** **2.** *Theorems 2 and 3 now hold when we add condition* (10) *to its assumptions.*

## 5. Final Comments

With reference to the graph domain setting, some questions and comments arise. We underline here that due to our approach we do not need to take the boundary into account, contrary to what is known from the literature. Should the graph became disjointed, we would obtain two different settings, and in fact two different problems would come under consideration. Toward the multiplicity of solutions, one may employ variational tools and critical point theory (see for example [15] for some tools available).

Now we turn the question of the Hadamard well-posedness of the given problem. To be more precise, let us formulate some background. Let *Z* be a metric space and let $\left(z_{n}\right)\subset Z$. We can consider the following family of problems for n∈N

(15) $$-\Delta_{s(x,\cdot )}u\left(x\right)+q\left(x\right){\left|u(x)\right|}^{p}u\left(x\right)=f\left(x,u\left(x\right),\nabla_{s(x,\cdot )}u\left(x\right),z_{n}\right)$$

with assumptions (independent on the parameter) leading to the existence and uniqueness of solutions employed in Section 3. By using the continuity of the solution operator in the uniqueness case as follows from the approach suggested in [16], we may obtain the Hadamard well-posedness, which says, roughly speaking, that small deviations from the unique solution return to such solution in the limit. Nevertheless, in case the solution is nonunique, we suggest some continuous dependence on parameters result as well. However, in this case we can approximate some solution with a subsequence only. Moreover, no procedure for how to obtain such subsequence is to be recovered, as suggested by the case of the Galerkin-type approximations in the non-unique case.

Another question concerns the structural stability, i.e., some changes introduced in the graph on which the problem is defined. Such a question is related to the above in case the weights are continuous, and we may proceed as sketched in the above remarks.

## Data Availability

Not applicable.
